# Supplementary material for: Development of a computer-aided design software for the quantitative evaluation of aesthetic damage
Source: PLoS One. 2019 Dec 18;14(12):e0226322. doi: 10.1371/journal.pone.0226322 (PMC6919621; doi:10.1371/journal.pone.0226322)
Supplement: S1 Code site — 1. (ZIP) [file pone.0226322.s001.zip › [EESC_jr][Projeto]Relatorio_07_21/vendors/select2/docs/_includes/examples/localization-rtl-diacritics.html]

# Localization, RTL and diacritics support

## Multiple languages

Select2 supports displaying the messages in different languages, as well
as providing your own
custom messages
that can be displayed.

The language does not have to be defined when Select2 is being
initialized, but instead can be defined in the `[lang]`
attribute of any parent elements as `[lang="es"]`.

{% highlight js linenos %}
$(".js-example-language").select2({
language: "es"
});
{% endhighlight %}

## RTL support

Select2 will work on RTL websites if the `dir` attribute is
set on the `<select>` or any parents of it. You can also
initialize Select2 with `dir: "rtl"` set.

{% highlight js linenos %}
$(".js-example-rtl").select2({
dir: "rtl"
});
{% endhighlight %}

## Diacritics support

Select2's default matcher will ignore diacritics, making it easier for
users to filter results in international selects. Type "aero" into the
select below.

Aeróbics
Aeróbics en Agua
Aerografía
Aeromodelaje
Águilas
Ajedrez
Ala Delta
Álbumes de Música
Alusivos
Análisis de Escritura a Mano

{% highlight js linenos %}
$(".js-example-diacritics").select2();
{% endhighlight %}
